# Unhealthy food consumption and its determinants among children aged 6 to 23 months in sub-Saharan Africa: a multilevel analysis of the demographic and health survey

**DOI:** 10.1186/s12887-023-04514-z

**Published:** 2024-01-13

**Authors:** Enyew Getaneh Mekonen, Alebachew Ferede Zegeye, Belayneh Shetie Workneh, Masresha Asmare Techane, Tadesse Tarik Tamir, Bewuketu Terefe

**Affiliations:** 1 Department of Surgical Nursing, School of Nursing, College of Medicine and Health Sciences, University of Gondar, Gondar, Ethiopia; 2 Department of Medical Nursing, School of Nursing, College of Medicine and Health Sciences, University of Gondar, Gondar, Ethiopia; 3 Department of Emergency and Critical Care Nursing, School of Nursing, College of Medicine and Health Sciences, University of Gondar, Gondar, Ethiopia; 4 Department of Pediatrics and Child Health Nursing, School of Nursing, College of Medicine and Health Sciences, University of Gondar, Gondar, Ethiopia; 5 Department of Community Health Nursing, School of Nursing, College of Medicine and Health Sciences, University of Gondar, Gondar, Ethiopia

**Keywords:** Unhealthy food consumption, Children, DHS, Sub-Saharan Africa, Multilevel analysis

## Abstract

**Background:**

Unhealthy food consumption that begins early in life is associated with a higher risk of nutrient inadequacy and related chronic diseases later in life. Healthy eating and consumption of important nutrients help to maintain a healthy body weight and reduce the risk of developing chronic conditions. Research from sub-Saharan Africa regarding consumption of unhealthy foods remains limited, with no studies quantifying the pooled prevalence among young children. Therefore, this study is intended to assess the pooled prevalence and determinants of unhealthy food consumption among children aged 6 to 23 months.

**Methods:**

Data from the most recent demographic and health surveys of five countries in sub-Saharan Africa conducted between 2015 and 2022 were used. A total weighted sample of 16,226 children aged 6 to 23 months was included in the study. Data extracted from DHS data sets were cleaned, recorded, and analyzed using STATA/SE version 14.0 statistical software. Multilevel mixed-effects logistic regression was used to determine the factors associated with the dependent variable. Intra-class correlation coefficient, likelihood ratio test, median odds ratio, and deviance (-2LLR) values were used for model comparison and fitness. Finally, variables with a p-value < 0.05 and an adjusted odds ratio with a 95% confidence interval were declared statistically significant.

**Results:**

The pooled prevalence of unhealthy food consumption among children aged 6 to 23 months was 13.41% (95% CI: 12.89-13.94%). Higher consumption of unhealthy foods was reported among mothers with low education [adjusted odds ratio (AOR) = 0.37; 95% confidence interval (CI) (0.30, 0.46)], unmarried women [AOR = 1.19; 95% CI (1.05, 1.34)], who had no media exposure [AOR = 0.64; 95% CI (0.56, 0.72)], delivered at home [AOR = 0.74; 95% CI (0.62, 0.87)], who hadn’t had a PNC checkup [AOR = 0.66; 95% CI (0.60, 0.73)], wealthier households [AOR = 1.20; 95% CI (1.05, 1.37)], older children (aged ≥ 9 months) [AOR = 3.88; 95% CI (3.25, 4.63)], and low community level media exposure [AOR = 1.18; 95% CI (1.04, 1.34)].

**Conclusion:**

Nearly one out of seven children aged 6 to 23 months consumed unhealthy foods. Maternal educational level, marital status of the mother, exposure to media, wealth index, place of delivery, PNC checkup, and the current age of the child were factors significantly associated with unhealthy food consumption. Therefore, improving women’s education, disseminating nutrition-related information through the media, providing more attention to poor and unmarried women, and strengthening health facility delivery and postnatal care services are recommended.

## Background

Dietary patterns are ever-changing towards higher consumption of added sugars, unhealthy fats, salt, and refined carbohydrates in various low- and middle-income countries (LMICs), including sub-Saharan Africa (SSA) [1]. Unhealthy food consumption (UFC) that begins early in life is associated with a higher risk of nutrient inadequacy, becoming overweight or obese, and related chronic diseases later in life [2]. Consumption of candies, chocolate, chips, French fries, cakes, and cookies displaces more nutritious foods and limits the consumption of crucial vitamins and minerals [3]. Repeated intake of unhealthy foods, including sweet beverages, enhances the need for the sweet taste, resulting in consumption of and a preference for sweet-tasting foods thereafter [2]. High consumption of unhealthy foods and beverages is also associated with poor diet quality among children [4].

Appropriate feeding in early childhood plays a central role in acceptable growth and development [5]. In the first 1000 days of life (from pregnancy to two years of age), nutritional status determines the body’s metabolic model, which often impacts the risk of juvenile and adult obesity and other diseases and lasts for a lifetime [6]. The nutritional factors in this time period include the nutritional status of the mother during pregnancy, breastfeeding, formula feeding, and appropriate complementary feeding [7]. The intake of solid or semi-solid foods increases at 6 months, whereas breastfeeding and formula feeding are progressively reduced [8].

The consumption of unhealthy foods are associated with higher risk of acquiring non-communicable diseases including type 2 diabetes, hypertension, hypercholesterolemia, and obesity [9]. Worldwide, around 43 million children aged 0–59 months are overweight or obese, and nearly 81% of them are living in LMICs [10]. Infant and young children’s feeding practices, rapid weight gain, the child’s diet, and a sedentary lifestyle are contributing factors to the development of infant and childhood obesity [11, 12]. Childhood overweight and obesity is attributed to the fifth leading global risk for mortality, accounting for 44% of the diabetes burden, 23% of the ischemic heart disease, and 7-41% of certain cancers [13]. Literature indicated an increasing burden of childhood overweight or obesity in SSA, with a pooled prevalence of 5.1%, and a higher prevalence was reported in the South African region (8.8%) [14, 15]. Unhealthy diets were also determinants of overweight or obesity among children and adolescents [16].

Healthy eating and consumption of important nutrients helps to achieve and maintain a healthy body weight and reduce the risk of developing high blood pressure, heart disease, type 2 diabetes, cancer, osteoporosis, iron deficiency, and dental caries [17]. Healthy eating behaviors are also associated with improved cognitive function [18]. Different guidelines indicated the need to avoid or limit unhealthy foods when feeding infant and young children [19]. However, research from the SSA regarding UFC remains limited, with no studies quantifying the pooled prevalence of UFC among young children in the region. To our knowledge, there is no study conducted in SSA to determine the pooled prevalence and associated factors of UFC among children aged 6 to 23 months using the most recent WHO and UNICEF guidelines for assessing infant and young child feeding practices published in 2021. Therefore, this study is intended to assess the pooled prevalence and determinants of unhealthy food consumption among children aged 6 to 23 months in sub-Saharan African countries using the recent demographic and health survey.

## Methods and materials

### Data sources, study design, and sampling

A cross-sectional pooled data using recent DHS from five sub-Saharan African countries, which were conducted between 2015/16 and 2022 was employed. Demographic and health surveys from five sub-Saharan African countries including Kenya (2022), Malawi (2015/16), Tanzania (2022), Uganda (2016), and South Africa (2016) were used. The data were appended to figure out the pooled prevalence of UFC and its determinants among children aged 6–23 months in sub-Saharan African countries. Different datasets, including those for children, males, women, births, and households are included in the survey for each country. For this study, the kid’s record (KR file) was used. The DHS is a nationwide survey mostly collected every five years across LMICs. It makes cross-country comparison possible as it uses standard procedures for sampling, questionnaires, data collection, cleaning, coding, and analysis [20].

The DHS employs a stratified two-stage sampling technique [21]. The first stage involves the development of a sampling frame, consisting of a list of primary sampling units (PSUs) or enumeration areas (EAs), which covers the entire country and is usually developed from the latest available national census. The second stage is the systematic sampling of households listed in each cluster or EA. In the current study, a total weighted sample of 16,226 children aged 6 to 23 months were (Table 1). Further information on the survey sampling strategies is available in the DHS guideline [22].

**Table 1:** Sample size for unhealthy food consumption and its determinants among children aged 6 to 23 months in sub-Saharan African countries

Country	Year of survey	Weighted sample (n)	Weighted sample (%)
Kenya	2022	2,944	18.14
Malawi	2015/16	4,843	29.85
Tanzania	2022	3,189	19.65
Uganda	2016	4,359	26.86
South Africa	2016	891	5.50
**Total sample size**		16,226	100

### Variables of the study

#### Outcome variable

The dependent variable of this study was unhealthy food consumption (“1”: consumed unhealthy food, “0”: didn’t consume unhealthy food). Unhealthy food consumption is defined as the consumption of selected sentinel foods (chocolates, sweets, candies, pastries, etc.) during the previous day [1]. If any amount of food from any of the sentinel categories has been consumed, children are counted as “consumed unhealthy food,” otherwise they are counted as “not consumed unhealthy food.”

#### Independent variables

**Fig. 1:**
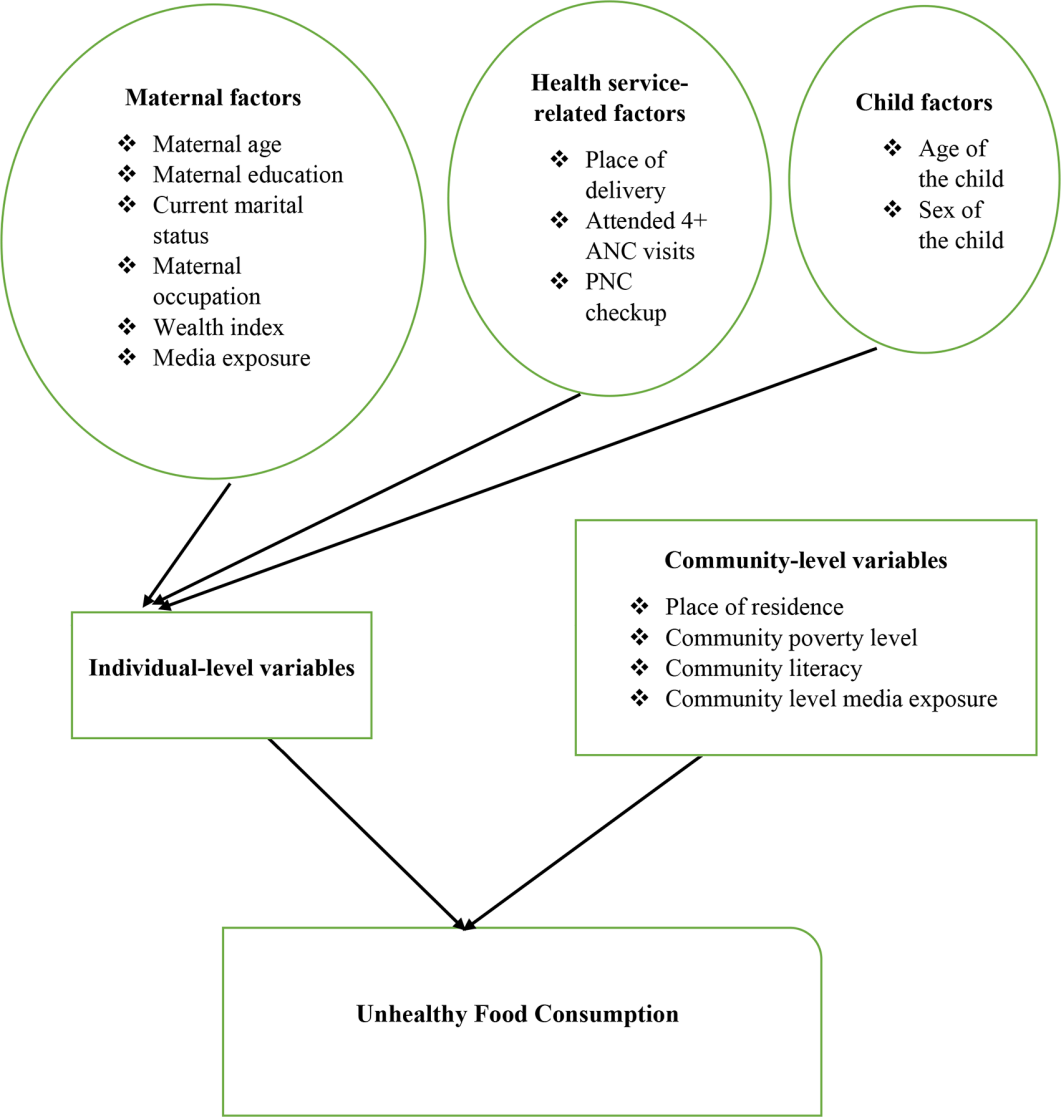
Conceptual framework for factors associated with unhealthy food consumption among children aged 6 to 23 months in SSA

Both individual and community-level variables were considered. Individual-level variables: maternal age (15–24 years, 25–34 years, 35–49 years), maternal education (no education, primary, secondary or higher), current marital status of the mother (married, unmarried), maternal occupation (not working, working), media exposure (yes, no), wealth index (poor, middle, rich), place of delivery (home, health facility), attended 4 + ANC visits (yes, no), PNC checkup (yes, no), age of the child (6–8 months, 9–11 months, 12–17 months, 18–23 months), and sex of the child (male, female). Community-level variables: place of residence (urban, rural), community poverty level (low, high), community literacy (low, high), and community level media exposure (low, high) (Fig. 1).

### Description of independent variables

#### Media exposure

Created by combining whether a respondent reads newspapers/magazines, listens to the radio, and watch television and coded as “yes” if the mother was exposed to at least one of these media and “no” otherwise.

#### Community level media exposure

The proportion of women who had exposed to at least one media; television, radio, or newspaper and categorized based on national median value as low (communities with ≤ 50% of women exposed) and high (communities with > 50% of women exposed) community-level media exposure.

#### Community literacy

The proportion of women with a minimum of primary level of education derived from data on respondents’ level of education. Then, it was categorized using national median value to values: low (communities with ≤ 50% of women have at least primary education) and high (communities with > 50% of women have at least primary education) community literacy.

#### Community poverty level

Aggregated variable from household wealth status (proportion of women from poor and rich wealth status) and it was recoded as low and high community poverty level likewise.

### Data processing and analysis

Data extracted from the recent DHS data sets were cleaned, recorded, and analyzed using STATA/SE version 14.0 statistical software. Sample weight was employed to manage sampling errors and non-responses. Continuous variables were categorized, and categorical variables were further re-categorized. Descriptive analysis was carried out to present the data in frequencies and percentages. Both the individual and community-level variables were presented using descriptive statistics. The DHS data’s variables were organized in clusters; 16,226 children are nested within households, and households were nested within 1692 clusters. The assumptions of independent observations and equal variance across clusters were broken to employ the traditional logistic regression model. This is an indication that using a sophisticated model to take into account between-cluster factors is necessary. As a result, multilevel mixed-effects logistic regression was used to determine the factors associated with UFC. Multilevel mixed effect logistic regression follows four models: the null model (outcome variable only), mode I (only individual-level variables), model II (only community-level variables), and model III (both individual and community-level variables). The model without independent variables (the null model) was used to check the variability of UFC across the cluster. The association of individual-level variables with the outcome variable (Model I) and the association of community-level variables with the outcome variable (Model II) were assessed. In the final model (Model III), the association of both individual and community-level variables was fitted simultaneously with the outcome variable (UFC).

The magnitude of the clustering effect and the degree to which community-level factors explain the unexplained variance of the null model were quantified by checking the intra-class correlation coefficient (ICC) and proportional change in variance (PCV). A model with the lowest deviance was selected as the best-fitted model. Finally, variables with a p-value less than 0.05 and an adjusted odds ratio (AOR) with a 95% confidence interval (CI) were described as statistically significant variables associated with the consumption of unhealthy foods. The presence of multi-collinearity between covariates was checked by using a variance inflation factor (VIF) falling within acceptable limits of 1–10, indicating the absence of significant collinearity across independent variables. Missing and “don’t know” data on foods and liquids given is treated as not given in numerator and included in denominator.

### Random effects

Random effects or measures of variation of the outcome variable were estimated using the median odds ratio (MOR), ICC, and PCV. ICC and PCV were used to measure the variation between clusters. Taking clusters as a random variable, the ICC reveals the variation of UFC between clusters and is computed as ICC = VC/(VC + 3.29)×100%. The MOR is the median value of the odds ratio between the area of the highest risk and the area of the lowest risk for UFC when two clusters are randomly selected, using clusters as a random variable; MOR = 𝑒 0.95√VC. In addition, the PCV demonstrates the variation in the prevalence of UFC explained by factors and computed as PCV= (Vnull-VC)/Vnull×100%; where Vnull = variance of the null model and VC = cluster level variance [23]. The association between the likelihood of unhealthy food consumption and individual and community-level independent variables was estimated by the fixed effects.

## Results

### Individual- and community-level characteristics of study subjects

A total of 16,226 children aged 6 to 23 months were included in this study. The mean age of mothers was 27.64 ± 0.05 years, and 43.61% of them fall in the age range of 25–34 years. More than half (53.39%) of the mothers completed primary education, and 81.84% of them are currently married. Regarding occupation, 64.10% of mothers had work, and nearly two-thirds (66.13%) of them had media exposure. More than one-third (33.93%) of the mothers in SSA had rich socioeconomic status. The majority (84.13%) of mothers in SSA countries were delivered at health facilities, and only 31.93% of them had PNC checkups. More than half (61.48%) of mothers attended 4 + ANC visits during their pregnancy. The mean age of children was 14.28 ± 0.04 months, and 33.16% of them fall in the age range of 12–17 months. More than half (50.95%) of children aged 6–23 months were male. More than three-fourths (76.16%) of the study subjects were from rural areas, and 54.36% of them had low community-level media exposure. More than half (50.44%) of mothers of children aged 6–23 months had low community-level poverty, and 60.48% of them had high community-level literacy (Table 2).

**Table 2:** Individual-and community-level characteristics of study subjects, pooled data from five SSA countries (*n* = 16,226)

Variables	Category	Frequency (n)	Percentage (%)
Maternal age	15–24 years	6,260	38.58
	25–34 years	7,076	43.61
	35–49 years	2,890	17.81
Maternal educational level	No formal education	2,364	14.57
	Primary	8,663	53.39
	Secondary and above	5,199	32.04
Current marital status of the mother	Married	13,280	81.84
	Unmarried	2,946	18.16
Maternal occupation	Not working	5,822	35.90
	Working	10,399	64.10
Exposure to media	Yes	10,731	66.13
	No	5,495	33.87
Wealth index	Poor	7,620	46.96
	Middle	3,100	19.11
	Rich	5,506	33.93
Place of delivery	Home	2,575	15.87
	Health facility	13,651	84.13
Attended 4 + ANC visits	Yes	9,976	61.48
	No	6,250	38.52
PNC checkup	Yes	4,993	31.93
	No	10,643	68.07
Age of child	6–8 months	2,809	17.32
	9–11 months	2,832	17.45
	12–17 months	5,381	33.16
	18–23 months	5,204	32.07
Sex of child	Male	8,267	50.95
	Female	7,959	49.05
Place of residence	Rural	12,357	76.16
	Urban	3,869	23.84
Community media exposure	Low	8,821	54.36
	High	7,405	45.64
Community poverty	Low	8,184	50.44
	High	8,042	49.56
Community literacy	Low	6,413	39.52
	High	9,813	60.48

### Pooled prevalence of unhealthy food consumption

**Fig. 2:**
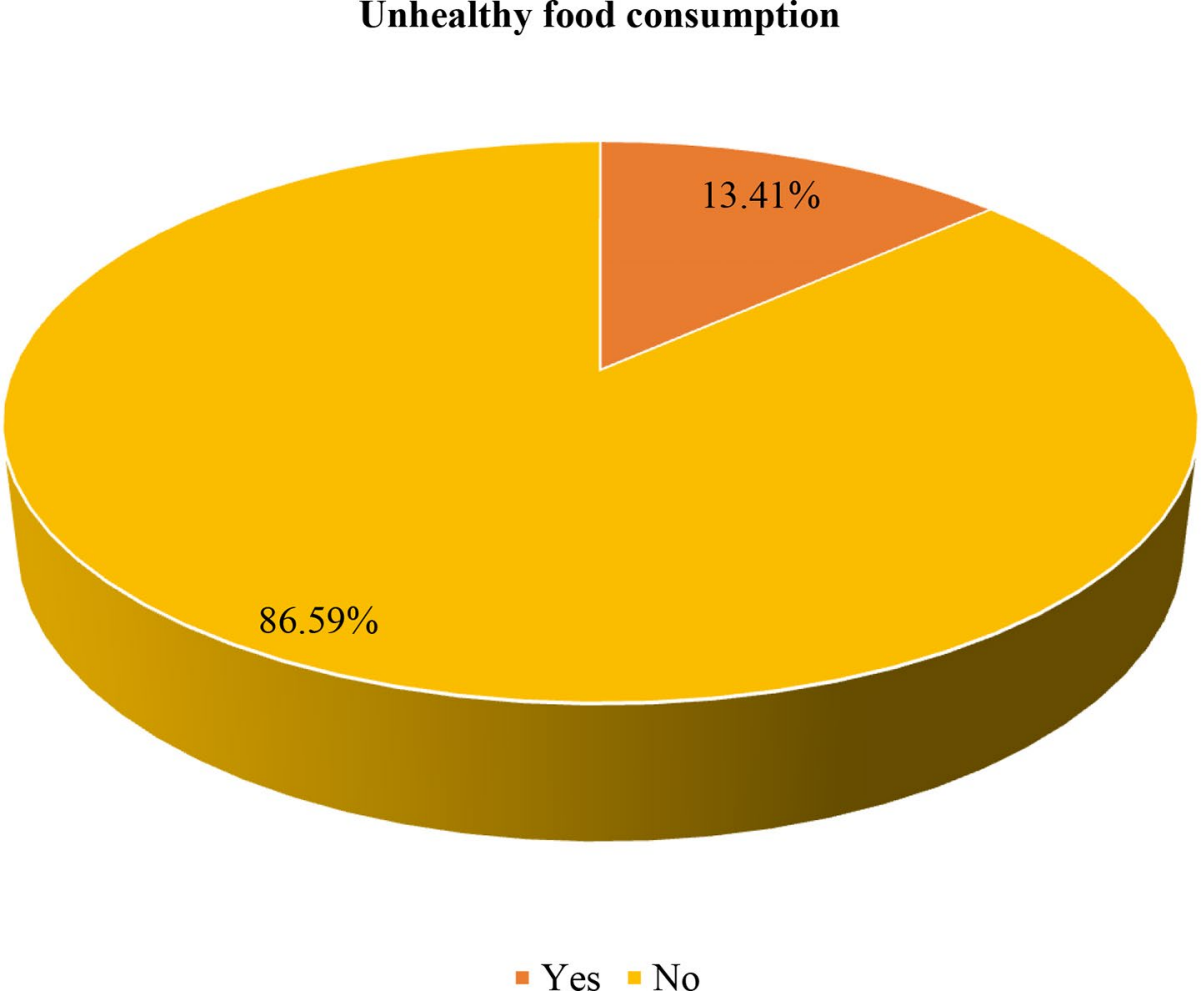
Unhealthy food consumption among children aged 6 to 23 months in sub-Saharan African countries, DHS 2015/16 to 2022 (*n* = 16,226)

**Fig. 3:**
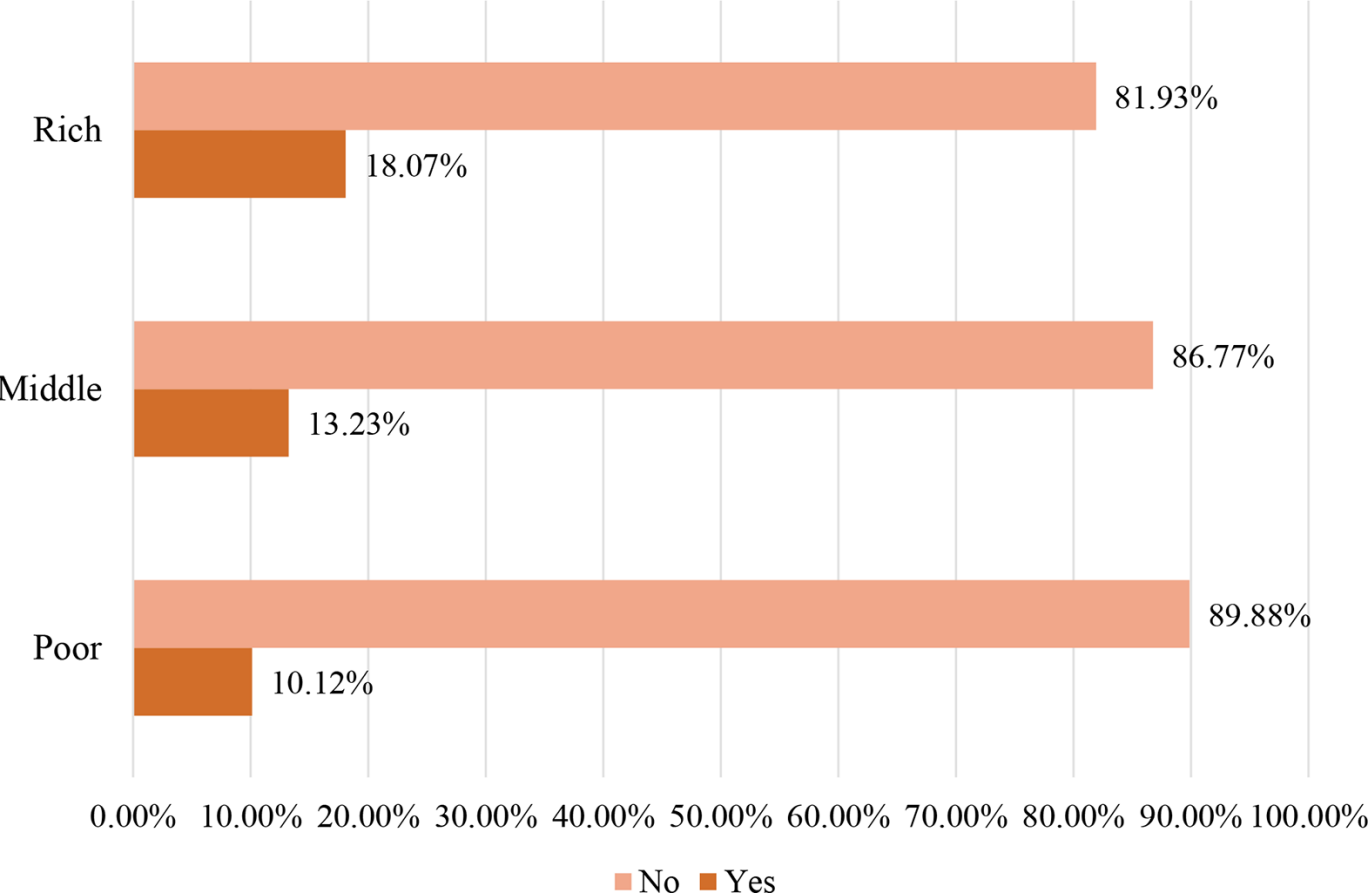
Unhealthy food consumption by household wealth status among children aged 6 to 23 months in sub-Saharan African countries, DHS 2015/16 to 2022 (*n* = 16,226)

**Fig. 4:**
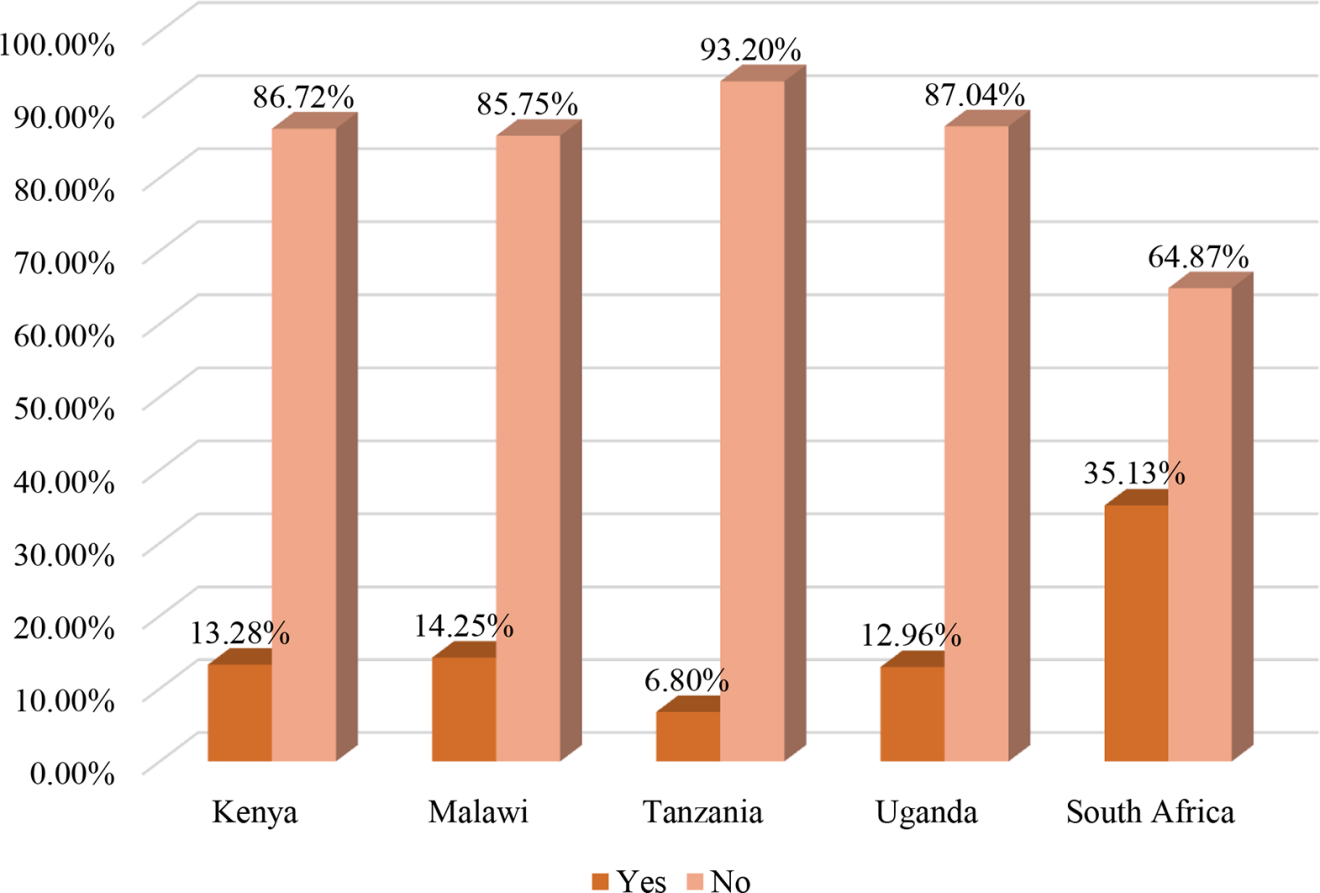
Unhealthy food consumption by country among children aged 6 to 23 months in sub-Saharan African countries, DHS 2015/16 to 2022 (*n* = 16,226)

In the present study, 13.41% (95% CI: 12.89–13.94%) of children aged 6–23 months consumed unhealthy foods during the day preceding the survey (Fig. 2). The result showed that consumption of unhealthy foods increases with increasing household wealth status, with the proportion of children who consumed unhealthy foods being lowest among children from poor household families (10.12%) and highest among rich household families (18.07%) (Fig. 3). The consumption of unhealthy foods was high in South Africa (35.13%), followed by Malawi (14.25%), and low in Tanzania (6.80%) (Fig. 4).

### Measures of variation and model fitness

A null model was used to determine whether the data supported the decision to assess randomness at the community level. Findings from the null model showed that there were significant differences in UFC between communities, with a variance of 0.2362062 and a P value of 0.000. The variance within clusters contributed 93.30% of the variation in UFC, while the variance across clusters was responsible for 6.70% of the variation. In the null model, the odds of UFC differed between higher- and lower-risk clusters by a factor of 1.52 times. The intra-class correlation value for Model I indicated that 5.70% of the variation in UFC accounts for the disparities between communities. Then, with the null model, we used community-level variables to generate Model II. According to the ICC value from Model II, cluster variations were the basis for 5.53% of the differences in UFC. In the final model (model III), which attributed approximately 5.61% of the variation in the likelihood of UFC to both individual and community-level variables, the likelihood of UFC varied by 1.49 times across low and high UFC (Table 3).

**Table 3:** Model comparison and random effect analysis for unhealthy food consumption among children aged 6–23 months in Sub-Saharan African countries

Parameter	Null model	Model I	Model II	Model III
Variance	0.2362062	0.199689	0.1924122	0.1955166
ICC	6.70%	5.70%	5.53%	5.61%
MOR	1.58	1.52	1.51	1.49
PCV	Reference	15.46%	18.54%	17.23%
**Model fitness**				
LLR	-6360.511	-5790.518	-6301.657	-5786.415
Deviance	12,721.022	11,581.036	12,603.314	11,572.830

ICC: Intra cluster correlation, LLR: log-likelihood ratio, MOR: median odds ratio, PCV: Proportional change in variance

### Individual and community-level factors associated with unhealthy food consumption

In the final fitted model of multivariable multilevel logistic regression, maternal educational level, marital status of the mother, exposure to media, wealth index, place of delivery, PNC checkup, the current age of the child, and community level media exposure were factors significantly associated with consumption of unhealthy foods among children aged 6–23 months. Mothers of children who completed secondary or higher education were 63% and 44% times less likely to give unhealthy foods to their child than those who had no education and completed primary education, respectively [AOR = 0.37; 95% CI (0.30, 0.46)] and [AOR = 0.56; 95% CI (0.50, 0.62)]. The odds of UFC were 1.19 times higher among unmarried women compared with their counterparts [AOR = 1.19; 95% CI (1.05, 1.34)]. Those mothers who had media exposure were 36% less likely to give their child unhealthy foods compared with mothers who hadn’t [AOR = 0.64; 95% CI (0.56, 0.72)].

Household wealth status was another determinant of UFC, in which children from wealthier households were 1.20 times more likely to consume unhealthy foods compared with those from poor economic status [AOR = 1.20; 95% CI (1.05, 1.37)]. Mothers who delivered at a health facility were 26% less likely to feed their child unhealthy foods compared with those who delivered at home [AOR = 0.74; 95% CI (0.62, 0.87)]. Children of mothers who had PNC checkups were 44% less likely to consume unhealthy foods compared with their counterparts [AOR = 0.66; 95% CI (0.60, 0.73)]. This study also revealed high consumption of unhealthy foods as the child gets older. The odds of UFC were 3.88, 2.80, and 2.00 times higher among children aged 18–23, 12–17, and 9–11 months compared with those aged 6–8 months [AOR = 3.88; 95% CI (3.25, 4.63)], [AOR = 2.80; 95% CI (2.34, 3.34)], and [AOR = 2.00; 95% CI (1.64, 2.45)]. Finally, children from a community with low media exposure were 1.18 times more likely to consume unhealthy foods compared with their counterparts [AOR = 1.18; 95% CI (1.04, 1.34)] (Table 4).

**Table 4:** Multivariable multilevel logistic regression analysis of individual and community-level factors associated with UFC among children aged 6–23 months in SSA countries

Variables	Category	Model I AOR (95% CI)	Model II AOR (95% CI)	Model III AOR (95% CI)
Maternal age	15–24 years	1.12 (0.97, 1.29)		1.12 (0.97, 1.30)
	25–34 years	1.01 (0.87, 1.15)		1.01 (0.87, 1.16)
	35–49 years	1		1
Maternal educational level	No formal education	0.36 (0.29, 0.45)*		0.37 (0.30, 0.46)*
	Primary	0.56 (0.50, 0.62)*		0.56 (0.50, 0.62)*
	Secondary and above	1		1
Marital status of the mother	Married	1		1
	Unmarried	1.19 (1.06, 1.34)*		1.19 (1.05, 1.34)*
Maternal occupation	Not working	0.91 (0.82, 1.01)		0.90 (0.81, 1.01)
	Working	1		1
Exposure to media	Yes	1		1
	No	0.65 (0.58, 0.74)*		0.64 (0.56, 0.72)*
Wealth index	Poor	1		1
	Middle	1.11 (0.96, 1.27)		1.09 (0.94, 1.25)
	Rich	1.25 (1.11, 1.41)*		1.20 (1.05, 1.37)*
Place of delivery	Home	0.73 (0.62, 0.86)*		0.74 (0.62, 0.87)*
	Health facility	1		1
Attended 4 + ANC visits	Yes	1		1
	No	0.92 (0.83, 1.02)		0.92 (0.83, 1.02)
PNC checkup	Yes	1		1
	No	0.66 (0.60, 0.73)*		0.66 (0.60, 0.73)*
Age of child	6–8 months	1		1
	9–11 months	2.01 (1.64, 2.45)*		2.00 (1.64, 2.45)*
	12–17 months	2.80 (2.34, 3.35)*		2.80 (2.34, 3.34)*
	18–23 months	3.88 (3.25, 4.63)*		3.88 (3.25, 4.63)*
Sex of child	Male	0.93 (0.84, 1.02)		0.93 (0.84, 1.02)
	Female	1		1
Place of residence	Rural		1	1
	Urban		1.56 (1.40, 1.74)*	1.07 (0.94, 1.21)
Community media exposure	Low		1.01 (0.90, 1.15)	1.18 (1.04, 1.34)*
	High		1	1
Community poverty	Low		1	1
	High		0.86 (0.76, 0.97)*	0.92 (0.81, 1.05)
Community literacy	Low		0.79 (0.69, 0.89)*	0.93 (0.81, 1.06)
	High		1	1

## Discussion

In the current study, the pooled prevalence of UFC among children aged 6–23 months in SSA countries was 13.41% (95% CI: 12.89–13.94%). This finding was lower than studies conducted in Ethiopia (63.7%) [24] and Brazil (43.1%) [25]. This discrepancy might be attributed to differences in sample size, study period, study area, and socio-economic status of the study subjects. The previous studies were conducted in a single area using a small sample size, whereas the current study uses pooled data from five countries and a larger sample size. The difference might also be due to differences in the determination of the outcome variable. The current study uses the 2021 WHO guideline indicators for infant and child feeding practices to get UFC. The study conducted in Brazil was conducted in 2019 before the declaration of this guideline.

Maternal educational status, marital status of the mother, exposure to media, wealth index, place of delivery, PNC checkup, the current age of the child, and community-level media exposure were determinants of UFC. Highly educated mothers were less likely to feed their children unhealthy foods. This finding was in agreement with studies conducted in Ethiopia [24], Brazil [25–27], and Mexico [28]. This might be due to the fact that mothers with a low educational level are more likely to have poor understandings about the significance of consuming healthy foods like fresh fruits and vegetables [29]. Mothers with a high educational level are also more often limited their children’s consumption of unhealthy foods such as sweets, soft drinks, and chips [30]. This can also be explained by the association between low maternal education and low purchasing capacity, as well as a lack of access to health-related information, which could possibly lead to the choice of unhealthy foods for children [31]. Thus, children of mothers with low education are better suited to be targeted for interventions aimed at reinforcing the attainment of healthy eating habits and reducing adverse health effects.

Similarly, the odds of UFC were 1.19 times higher among unmarried women compared with their counterparts. This might be due to the lower average nutritional status of never-married, widowed, and divorced women [32]. This can also be explained by the fact that married mothers living with their husbands are more knowledgeable about healthy foods for children, and husbands may contribute money to buy different foods. Mothers are better encouraged to stay in relationships to get the most basic support from their partner. Mothers who had media exposure were less likely to give their children unhealthy foods compared with mothers who hadn’t. Likewise, children from a community with low media exposure were 1.18 times more likely to consume unhealthy foods compared with their counterparts. This finding was supported by a study conducted in Indonesia [33]. This might be due to the effectiveness of mass media in disseminating healthy feeding messages, as large audiences across boundaries can be potentially reached [34]. Mother’s knowledge, attitudes, beliefs, and behaviors towards healthy eating habits can be shaped through routine exposure to mass media [35]. Mass media campaigns for health promotion and disease prevention, including healthy eating habits among children, are essential to reducing unhealthy food consumption.

Children from wealthier households were 1.20 times more likely to consume unhealthy foods compared with those from poor wealth status. A similar finding was reported by a study conducted in West Africa [36]. This might be attributed to the fact that the richest families may have the income to buy unhealthy foods like sweets and chocolates. Children from wealthier households may also consume candies, chocolate, chips, French fries, cakes, and cookies more than those from poor families, as they rely on other cheap foods. Children of mothers who delivered at a health facility and had a PNC checkup were less likely to consume unhealthy foods. This might be due to the fact that mothers who deliver at health facilities can get advice from health professionals about healthy feeding habits. The counseling provided to mothers during post-natal checkups could also contribute to decreased consumption of unhealthy foods. Finally, high consumption of unhealthy foods was reported as the child got older. The odds of UFC were four, three, and two times higher among children aged 18–23, 12–17, and 9–11 months compared with those aged 6–8 months. This finding was consistent with studies conducted in African and Asian urban contexts [37], Nepal [38], and West Africa [36]. Our finding was contradicted by a study conducted in Ethiopia [24]. This might be due to the fact that as the child gets older, they become more vulnerable to the effects of different situations they encounter, enabling food selections that are not healthy. Another possible reason could be child preference and a strong claim for sweet and inconvenient foods as they get older.

### Strengths and limitations of the study

The use of a nationally representative, large sample size across three countries in SSA to determine the pooled prevalence of UFC and identify its individual and community-level factors among children aged 6 to 23 months is the main strength of this study. The other strength of the present study is the use of advanced statistical models that consider individual and community-level factors. The current study also has some limitations. First, as we only included five countries in the SSA, the findings may not be generalizable to all SSA countries. Secondly, the causal relationship between the outcome variable and independent variables could not be established due to the cross-sectional nature of the study design. Finally, there might be a possibility of recall bias as the DHS survey depends on respondents’ self-reporting.

## Conclusion

Nearly one out of seven children aged 6 to 23 months consumed unhealthy foods. Maternal educational level, marital status of the mother, exposure to media, wealth index, place of delivery, PNC checkup, and the current age of the child were factors significantly associated with unhealthy food consumption. Therefore, improving women’s education, disseminating nutrition-related information through the media, providing more attention to poor and unmarried women, and strengthening health facility delivery and postnatal care services are recommended to reduce the consumption of unhealthy foods by infants and young children.

## Data Availability

The data from the five SSA countries is publicly available online at (https://dhsprogram.com/data/available-datasets.cfm).

## Abbreviations

ANC: Antenatal Care
AOR: Adjusted Odds Ratio
CI: Confidence Interval
DHS: Demographic and Health Survey
ICC: Intra-class Correlation Coefficient
IYC: Infants and Young Children
LMICs: low- and middle-income countries
MOR: Median Odds Ratio
PCV: Proportional Change in Deviance
PNC: Postnatal Care
SSA: sub-Saharan Africa
UFC: Unhealthy Food Consumption
VIF: Variance Inflation Factor
WHO: World Health Organization
